# An analysis of factors influencing cognitive dysfunction among older adults in Northwest China based on logistic regression and decision tree modelling

**DOI:** 10.1186/s12877-024-05024-y

**Published:** 2024-05-07

**Authors:** Yu Wang, Li Dou, Ni Wang, Yanjie Zhao, Yuqin Nie

**Affiliations:** 1 Zhejiang Provincial People’s Hospital, No.158 Shangtang Road, Hangzhou City, Zhejiang Province 310014 People’s Republic of China; 2 The Second Affiliated Hospital of Xinjiang Medical University, No. 38, North 2nd Lane, Nanhu East Road, Shuimogou District, Urumqi City, Xinjiang Uygur Autonomous Region 830063 People’s Republic of China; 3 School of Nursing, Xinjiang Medical University, No.567 Shangde North Road, Urumqi City, Xinjiang Uygur Autonomous Region 830000 People’s Republic of China

**Keywords:** Cognitive dysfunction, Risk factors, Decision tree, Older people

## Abstract

**Background:**

Cognitive dysfunction is one of the leading causes of disability and dependence in older adults and is a major economic burden on the public health system. The aim of this study was to investigate the risk factors for cognitive dysfunction and their predictive value in older adults in Northwest China.

**Methods:**

A cross-sectional study was conducted using a multistage sampling method. The questionnaires were distributed through the Elderly Disability Monitoring Platform to older adults aged 60 years and above in Northwest China, who were divided into cognitive dysfunction and normal cognitive function groups. In addition to univariate analyses, logistic regression and decision tree modelling were used to construct a model to identify factors that can predict the occurrence of cognitive dysfunction in older adults.

**Results:**

A total of 12,494 valid questionnaires were collected, including 2617 from participants in the cognitive dysfunction group and 9877 from participants in the normal cognitive function group. Univariate analysis revealed that ethnicity, BMI, age, educational attainment, marital status, type of residence, residency status, current work status, main economic source, type of chronic disease, long-term use of medication, alcohol consumption, participation in social activities, exercise status, social support, total scores on the Balanced Test Assessment, total scores on the Gait Speed Assessment total score, and activities of daily living (ADL) were significantly different between the two groups (all *P* < 0.05). According to logistic regression analyses, ethnicity, BMI, educational attainment, marital status, residency, main source of income, chronic diseases, annual medical examination, alcohol consumption, exercise status, total scores on the Balanced Test Assessment, and activities of daily living (ADLs) were found to influence cognitive dysfunction in older adults (all *P* < 0.05). In the decision tree model, the ability to perform activities of daily living was the root node, followed by total scores on the Balanced Test Assessment, marital status, educational attainment, age, annual medical examination, and ethnicity.

**Conclusions:**

Traditional risk factors (including BMI, literacy, and alcohol consumption) and potentially modifiable risk factors (including balance function, ability to care for oneself in daily life, and widowhood) have a significant impact on the increased risk of cognitive dysfunction in older adults in Northwest China. The use of decision tree models can help health care workers better assess cognitive function in older adults and develop personalized interventions. Further research could help to gain insight into the mechanisms of cognitive dysfunction and provide new avenues for prevention and intervention.

## Introduction

The pathological stages of cognitive impairment range from mild cognitive dysfunction to dementia [1], and its main clinical manifestation in patients is a decline in memory function. A study predicted that there would be 83.2 million cases of cognitive impairment among elderly individuals worldwide by 2030 [2]. The prevalence of cognitive dysfunction in China was as high as 15.54% in 2020, accounting for a total of 38.77 million people [3]. One meta-analysis reported that the pooled prevalence of dementia was highest in western China (9.6%), intermediate in northern China (5.4%), and lowest in central (3.8%) and southern China (3.7%) [4]. Cognitive dysfunction in older adults is affected by a variety of factors, such as age, social interaction, mental health (PWB), lifestyle, personality traits, and indoor air pollution exposure [5–7]. Studying the factors influencing cognitive functioning in older adults is important for preventing and managing cognitive impairment diseases and improving the health and quality of life of older adults in terms of cognitive functioning. Therefore, logistic regression analysis models are often used in combination with decision tree models to improve analytical efficacy [8, 9].

Despite important advances in the study of cognitive dysfunction in older adults, there is still a need for further exploration of the factors influencing cognitive dysfunction in older adults globally. For example, the sample sizes in some studies are relatively small and may not be sufficiently diverse to represent the entire population of older adults [10]. There are limitations in terms of sample size when using decision tree modelling, thus potentially limiting the generalizability of the findings. In addition, previous studies have not considered a wider range of potential influencing factors in the modelling process, which may affect the predictive accuracy of the model [11]. In the present study, with sufficient samples, the inclusion of indicators was more comprehensive not only for the inclusion of potential risk factors for cognitive functioning in older adults but also for the inclusion of traditional risk factors, such as balance test, gait speed assessment, and physical functioning assessment scales (e.g., self-care in daily life). In addition, the factors influencing cognitive dysfunction in older adults may vary across cultural backgrounds and geographic regions. Therefore, cross-cultural research on a global scale is crucial for a more comprehensive understanding of the factors influencing cognitive dysfunction in older adults. The purpose of this study was to analyse the factors affecting cognitive dysfunction in older people with different characteristics through the joint use of a decision tree model and a logistic regression model. This study provides a reference for meeting the differentiated and diversified service needs of elderly people and for health care professionals to develop targeted interventions for cognitive dysfunction in elderly people in Xinjiang.

## Materials and methods

### Research design

In this study, data from 8 tertiary hospitals, 9 secondary hospitals, 7 community health service centres and 8 elderly care facilities in 4 regions (the southern Xinjiang region, northern Xinjiang region, eastern Xinjiang region and Uchang region) of the Xinjiang Uygur Autonomous Region of China were selected for cross-sectional study using a multistage sampling method.

### Participants

The Elderly Disability Monitoring Platform (EDMP) is a platform for older people aged 60 years and above in China. The platform was used by researchers to collect detailed demographic information and conduct cross-sectional surveys on the five dimensions of vision, hearing, walking, sound composition and cognition among all older adults. Participants recruited between 6 December 2021 and 8 June 2023 were included in this study. The inclusion criteria were as follows: ① ≥ 60 years of age and ② good communication skills. The exclusion criteria were as follows: ① suffering from schizophrenia, depression or other mental illnesses; ② unable to cooperate with the physical function survey (e.g., finished); ① suffering from schizophrenia, depression or other mental illnesses; ② inability to cooperate with the physical function survey (e.g., completely disabled); and ③ experiencing the acute stage of illness (e.g., surgery).

### Ethical principles

The study was based on the principles of the Declaration of Helsinki. All patients provided informed consent, and the study was approved by the Beijing Hospital Ethics Committee (2021BJYYEC-325-01).

### Research tools

#### General information questionnaire

This questionnaire assessed sex, height, weight, date of birth, ethnicity, religion, education level, marital status, number of children, type of residence, current employment status, type of health insurance, monthly household income, main source of income, types of chronic diseases, types of long-term medications used, alcohol consumption, smoking status, annual medical check-ups, participation in social activities, physical exercise, and social support. Body Mass Index (BMI) is a common international standard for measuring the degree of fatness and thinness of the human body and whether it is healthy or not. The formula is: BMI = weight/ height^2^ (weight in kilograms; height in meters).According to WHO’s BMI classification criteria, BMI ≥ 28 (obese), 24 ≤ BMI < 28 (overweight), 18.5 ≤ BMI < 24 (normal), < 18.5 (peak).

#### Cognitive function

Cognitive function was assessed using the Mini-Mental State Examination (MMSE), which was developed by Folstein et al. [12] in 1975 and consists of items regarding time and place orientation, immediate memory and recall, attention and calculation, and language and visuospatial structural abilities, with a total possible score of 30 points. The higher the score is, the better an individual’s cognitive functioning. Normal cognitive function is indicated by scores > 24 points (secondary school education and above), > 20 points (primary school education) and > 17 points (illiterate); otherwise, an individual is considered to have cognitive dysfunction. The Chinese version of the MMSE was developed by Wang Zhengyu et al. [13] in 1989, with a test-retest reliability of 0.91.

#### Ability to perform activities of daily living

Basic activities of daily living (BADL) and instrumental activities of daily living (IADL) are included in activities of daily living. The ability to perform BADL was evaluated by the Barthel Index (BI), a scale constructed by Mahoney and Barthel [14] in 1965 that consists of 10 items. The scale has a total possible score of 100, with higher scores indicating a greater ability to perform BADL. A total score of < 40 was classified as severe dependence, a score of 41–60 was classified as moderate dependence, a score of 61–99 was classified as mild dependence, and a score of 100 was classified as no dependence. The ability to perform IADL was measured by the Instrumental Activities of Daily Living (IADL) Competence Scale, constructed by Lawton et al. [15] in 1965, which contains 8 items and has a total possible score of 0–24; higher scores indicate a greater ability to perform IADL. A total score of 24 was considered to indicate no ability. A total score of 24 was considered to indicate a lack of IADL dependence; otherwise, the score was considered IADL dependent.

#### Balance

 Balance, walking speed and muscle strength were assessed using the Short Physical Performance Battery (SPPB), which was developed by the National Center on Aging at [16] and consists of three dimensions to measure balance, walking speed and muscle strength in older adults. The balance test consisted of two-legged combined standing, semi-anterior-posterior standing, and anterior-posterior standing, with two-legged combined standing and semi-anterior-posterior standing scoring as follows: 1 point for > 10 s, 1 point for 3 to < 10 s, and 2 points for 10 s. The step speed test was a 2.44-m walking speed test, scored as follows: 1 point for a speed < 0.43 m/s, 2 points for 0.44 to 0.60 m/s, 3 points for 0.61 to 0.77 m/s, and 4 points for ≥ 0.78 m/s. Plyometric testing was performed in 5 sit-to-stand trials, with 1 point given for 16.70–60 s, 2 points for 13.70–16.69 s, 3 points for 11.20–13.69 s, and 4 points for ≤ 11.19 s. The total score for each dimension ranged from 0 to 4, with higher scores corresponding to better function, and a total score < 3 was classified as abnormal function [17].

### Statistical analysis

Statistical analysis was performed using SPSS 24.0 software. Frequency counts, constitutive ratios, means and standard deviations were used for descriptive statistics of general information. A logistic regression analysis model and a decision tree model were also established to compare the influencing factors of the two models. The decision tree model was analysed using the classification and regression tree (CART) algorithm, which included all independent variables in the decision tree model and set the minimum number of cases of the parent node and child node in the parameter design to 400 and 100, respectively, and the test level of splitting and merging at α = 0.05. For the logistic regression model, the presence or absence of cognitive impairment was used as the dependent variable, and the independent variables that were statistically significant in the one-way outcome analysis were used to establish the model, with *P* < 0.05 indicating a statistically significant difference. For the overall results and evaluation of the two models, the Hosmer‒Lemeshow goodness-of-fit test was used, and the overall correctness of the prediction, the model risk statistic, and the subject operating characteristic curve (receiver operating characteristic curve (ROC), specificity, sensitivity, and Jordon’s index) were also used.

## Results and discussion

### Results

#### Description of each indicator

A total of 12,494 questionnaires were distributed in this study, and 12,494 valid questionnaires were recovered, thus yielding a valid recovery rate of 100%. There were 2,617 patients (20.95%) in the cognitive dysfunction group and 9,877 patients (79.05%) in the normal cognitive function group.

#### Comparison of the occurrence of cognitive dysfunction in older people with different characteristics

Based on whether cognitive dysfunction occurred, the participants were divided into 2 groups: the cognitive dysfunction group (*n* = 2617) and the normal cognitive function group (*n* = 9877). The general information and physical and cognitive functions of the 2 groups were compared. There were no statistically significant differences in the occurrence of cognitive dysfunction based on sex or smoking status (*P* > 0.05). There were differences in the occurrence of chronic diseases among participants with different ethnicities, BMIs, ages, educational attainment statuses, marital statuses, types of residence, residency, current work status, main economic sources, types of chronic diseases, long-term use of medications, alcohol consumption, participation in social activities, exercise status, social support status, total scores on the Balanced Test Assessment, total scores on the Gait Speed Assessment, and activities of daily living (ADLs)(*P* < 0.05), as shown in Table 1.

**Table 1:** Comparison of the occurrence of cognitive dysfunction in older adults with different characteristics

Variable	Cognitive dysfunction group (*n* = 2617)	Normal cognitive function group (*n* = 9877)	χ2	*P*
**BMI**			54.953	< 0.001
Peak	170(6.5%)	351(3.6%)		
Normal	1143(43.7%)	4136(41.9%)		
Overweight (baggage, freight)	963(36.8%)	3880(39.3%)		
Obese	341(13.0%)	1510(15.3%)		
**Ethnicity**			73.034	< 0.001
Han ethnic group	1475(56.4%)	6177(62.5%)		
Other	63(2.4%)	358(3.6%)		
Hui ethnic group	71(2.7%)	360(3.6%)		
Kazakh ethnic group	98(3.7%)	320(3.2%)		
Uighur ethnic group	910(34.8%)	2662(27.0%)		
**Age**	2617(21.2%)	9817(78.8%)	65.817	< 0.001
**Educational attainment**			123.320	< 0.001
Illiteracy	11(0.4%)	93(0.9%)		
Secondary school	1493(57.1%)	5013(50.8%)		
Junior high school	740(28.3%)	2426(24.6%)		
High school or secondary school	267(10.2%)	1534(15.5%)		
University college	81(3.1%)	615(62%)		
Undergraduate and above	25(1.0%)	196(2.0%)		
**Marital status**			323.415	< 0.001
Unmarried	19(0.7%)	22(0.2%)		
Married	1928(73.7%)	8570(86.8%)		
Divorced	35(1.3%)	111(1.1%)		
Bereaved (literary)	620(23.7%)	1061(10.7%)		
Other	15(0.6%)	1131.1%)		
**Type of residence**			27.094	< 0.001
Rural	1708(65.3%)	6967(70.5%)		
Urban	909(34.7%)	2910(29.5%)		
**Residency**			83.830	< 0.001
Living alone	146(5.6%)	558(5.6%)		
Living with family	2403(91.8%)	9261(93.8%)		
Institutions for elderly care	68(2.6%)	58(0.6%)		
**Current work status**			8.206	0.042
Retired (from work)	34(1.3%)	184(1.9%)		
Farming	56(2.1%)	153(1.5%)		
Incumbency	2025(77.4%)	7615(77.1%)		
Profession	502(19.2%)	1925(19.5%)		
**Main economic source**			187.916	< 0.001
Without financial resources	88(3.4%)	363(3.7%)		
Retirement pay or pension	1485(56.7%)	6546(66.3%)		
From children	608(23.2%)	1329(13.5%)		
Income from low income	188(7.2%)	479(4.8%)		
Income from labour	248(9.5%)	1160(11.7%)		
**Chronic diseases**			111.178	< 0.001
Does not have	353(13.5%)	2264(22.9%)		
Does have	2264(86.5%)	7613(77.1%)		
**Long-term use of medications**			64.003	< 0.001
Does not have	734(28.0%)	3597(36.4%)		
Does have	1883(72.0%)	6280(63.6%)		
**Alcohol consumption**			16.034	< 0.001
Never	1918(73.3%)	7099(71.9%)		
Used to drink but quit	570(21.8%)	2075(21.0%)		
All the time	129(4.9%)	703(7.1%)		
**Whether annual medical examination**			101.95	< 0.001
No	912(34.8%)	2468(25.0%)		
Yes	1705(65.2%)	7409(75.0%)		
**Participation in social activities**			11.427	0.010
No	2586(98.8%)	9685(98.1%)		
1–3 days per week	25(1.0%)	109(1.1%)		
4–6 days per week	4(0.2%)	50(0.5%)		
Daily participation	2(0.1%)	33(0.3%)		
**Whether or not you exercise**			338.575	< 0.001
No	1228(46.9%)	2808(28.4%)		
1–3 days per week	683(26.1%)	3048(30.9%)		
4–6 days per week	246(9.4%)	1328(13.4%)		
Daily participation	460(17.6%)	2693(27.3%)		
**Social support**			152.061	< 0.001
Does not have	279(10.7%)	585(5.9%)		
Emotional support only	119(4.5%)	300(3.0%)		
Material support only	139(5.3%)	250(2.5%)		
Both	2080(79.5%)	8742(88.5%)		
**Total scores on the Balanced Test Assessment**	2617(21.2%)	9817(78.8%)	-31.917	< 0.001
**Total scores on the Gait Speed Assessment**	2617(21.2%)	9817(78.8%)	-20.935	< 0.001
**Activities of daily living (ADLs)**	2617(21.2%)	9817(78.8%)	2646.954	< 0.001

##### Logistic regression analysis

When cognitive dysfunction was used as the dependent variable (no = 0, yes = 1), the following 18 factors were statistically significant in the one-way analyses and were subsequently entered into the two-way logistic regression model as independent variables: ethnicity, BMI, age, educational attainment, marital status, type of residence, residency, current work status, main economic sources, type of chronic disease, long-term use of medications, alcohol consumption, participation in social activities, whether or not one exercised, social support, total scores on the Balanced Test Assessment, total scores on the Gait Speed Assessment, and activities of daily living (ADLs). Binary logistic regression revealed that the following variables were risk factors for cognitive dysfunction in older adults: nationality, BMI, educational attainment, marital status, residency, main source of income, chronic diseases, annual medical examination, alcohol consumption, exercise status, total scores on the balanced test assessment, and activities of daily living (ADL) (*P* < 0.05). The results are shown in Table 2.

**Table 2:** Dichotomous logistic regression analysis of factors influencing cognitive dysfunction

Variable	B	SE	Wald χ2	*P*	OR	95% CI	
						Lower limit	Upper limit
Constant	-0.76	0.508	0.023	0.880	0.926		
**BMI**			8.519	0.036			
Normal	0.349	0.131	7.056	0.008	1.418	1.096	1.834
Overweight (baggage, freight)	0.180	0.079	5.234	0.022	1.197	1.026	1.396
Obese	0.159	0.079	4.071	0.044	1.172	1.005	1.367
**Age**	0.014	0.002	51.810	< 0.001	1.014	1.010	1.018
**Ethnicity**			22.104	< 0.001			
Other	-0.200	0.064	9.682	0.002	0.818	0.721	0.929
Hui ethnic group	-0.641	0.161	15.929	< 0.001	0.527	0.385	0.722
Kazakh ethnic group	-0.249	0.148	2.840	0.092	0.779	0.583	1.041
Uighur ethnic group	-0.004	0.137	0.001	0.975	0.996	0.762	1.301
**Educational attainment**			109.138	< 0.001			
Secondary school	-0.495	0.435	1.295	0.255	0.610	0.260	1.429
Junior high school	0.258	0.247	1.095	0.295	1.294	0.798	2.099
High school or secondary school	0.835	0.247	11.429	< 0.001	2.305	1.420	3.741
University college	0.325	0.253	1.653	0.199	1.384	0.843	2.2712
Undergraduate and above	0.163	0.273	0.357	0.550	1.177	0.689	2.012
**Marital status**			61.220	< 0.001			
Married	1.502	0.505	8.840	0.003	4.490	1.668	12.083
Divorced	0.404	0.325	1.541	0.215	1.497	0.792	2.831
Bereaved (literary)	0.786	0.393	3.994	0.046	2.195	1.015	4.744
Other	0.926	0.329	7.898	0.005	2.523	1.323	4.811
Type of residence	0.241	0.068	12.632	< 0.001	1.272	1.114	1.453
**Residency**			11.900	0.003			
Living with family	-0.878	0.255	11.808	< 0.001	0.416	0.252	0.686
Institutions for elderly care	-0.704	0.240	8.587	0.003	0.495	0.309	0.792
**Main source of income**			39.784	< 0.001			
Without financial resources	-0.307	0.161	3.647	0.056	0.736	0.537	1.008
From children	-0.202	0.092	4.831	0.028	0.817	0.682	0.978
Income from low income	0.244	0.097	6.384	0.012	1.277	1.056	1.544
Income from labour	-0.033	0.126	0.070	0.791	0.967	0.755	1.238
**Chronic diseases**	-0.405	0.707	33.890	< 0.001	0.667	0.582	0.765
**Whether annual medical examination**	0.318	0.057	31.457	< 0.001	1.374	1.230	1.536
**Alcohol consumption**			7.287	0.026			
Used to drink but quit	0.267	0.111	5.839	0.016	1.306	1.052	1.623
All the time	0.322	0.120	7.232	0.007	1.380	1.091	1.746
**Whether or not you exercise**			9.095	0.028			
1–3 days per week	0.029	0.074	0.155	0.694	1.029	0.891	1.189
4–6 days per week	-0.084	0.073	1.352	0.245	0.919	0.797	1.060
Daily participation	-0.211	0.095	5.001	0.025	0.810	0.673	0.974
**Total scores on the Balanced Test Assessment**	-0.210	0.015	189.735	< 0.001	0.810	0.787	0.835
**Activities of Daily Living (ADLs)**	-0.029	0.001	626.743	< 0.001	0.972	0.969	0.974

##### Decision tree modelling analysis of factors influencing cognitive dysfunction in community-dwelling older adults

**Fig. 1:**
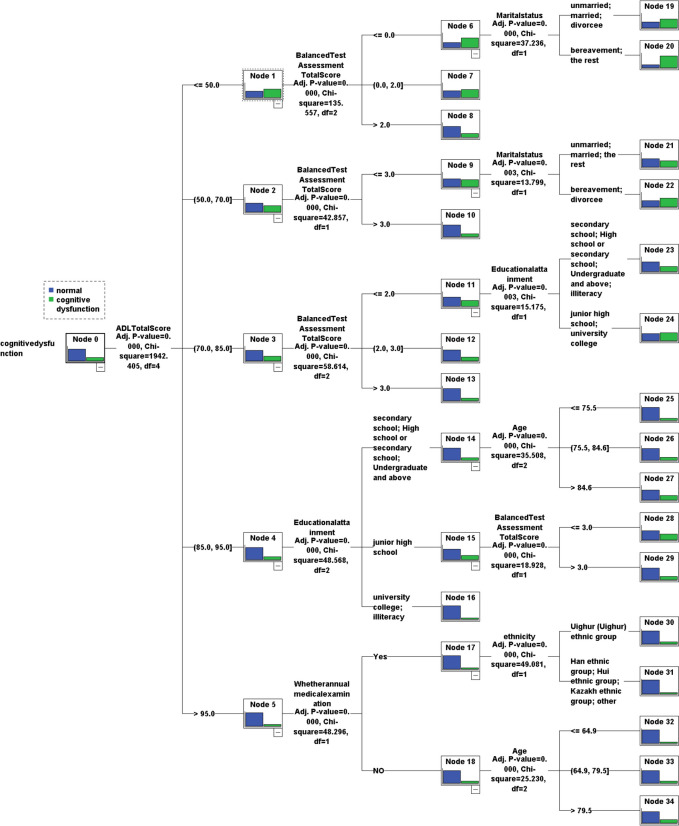
Decision tree model analysis of factors influencing cognitive dysfunction

The chi-square automatic interaction detection (CHAID) algorithm was used, and the significance level for decision tree growing branch splitting was 0.05. The minimum sample size of the parent node was set to 400, and the minimum sample size of the child node was set to 100. If the sample size on the node did not meet this requirement, the node was considered the terminal node, and no further splitting was performed. Variables that were statistically significant in the dichotomous logistic regression analysis were included. In this study, a decision tree was constructed at 3 levels with 22 terminal nodes, and 6 explanatory variables were screened: activities of daily living (ADL), total scores on the balanced test assessment, educational attainment, marital status, age, ethnicity, and annual medical examination, as shown in Fig. 1.

##### Comparison of the decision tree model and logistic regression model

**Fig. 2:**
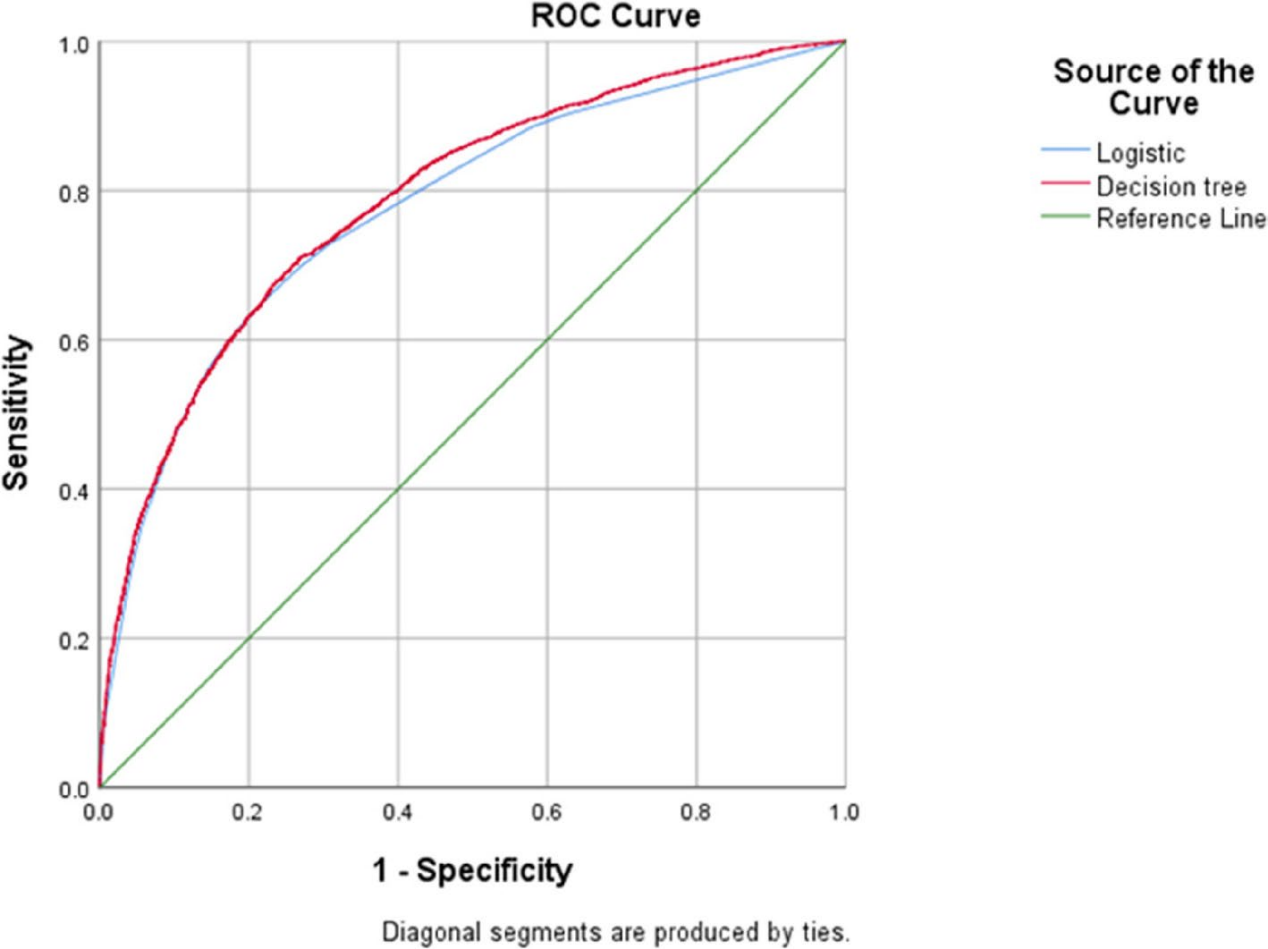
ROC curves for the logistic regression and decision tree models

The ROC curves were plotted based on the influencing factor models established by logistic regression and decision tree modelling (Fig. 2). The area under the ROC curve for the logistic regression model was 0.778 (95% CI: 0.765–0.787), with a sensitivity of 0.709 and a specificity of 0.733. The area under the ROC curve for the decision tree model was 0.788 (95% CI: 0.778–0.798), with a sensitivity of 0.656 and a specificity of 0.776. The difference in the area under the ROC curve of the two models was not statistically significant (Z = 1.414, *P* > 0.05), and the predictive effects were similar (see Table 3).

**Table 3:** Comparison of the classification effects of the logistic regression and decision tree models

Model	AUC	SE	*P*	95% CI
Logistic regression	0.778	0.005	0.000	0.765–0.787
Decision tree	0.788	0.005	0.000	0.778–0.798

## Discussion

Our study aimed to provide reliable evidence on the factors influencing the risk of developing cognitive dysfunction in older adults in Northwest China. Compared with previous studies, in our study, we not only used traditional statistical analyses but also introduced decision tree analysis to enhance the reliability of our results.

### Impact of potentially modifiable risk factors on cognitive dysfunction in older adults

BMI and alcohol consumption are well-known potentially modifiable risk factors affecting cognitive dysfunction in older adults [18], and the results of the present study are generally consistent with those of previous studies. The results of the data analysis in this study showed that there were significant differences between the cognitive dysfunction group and the normal cognitive function group in terms of BMI, literacy level, and alcohol consumption. Accordingly, both logistic regression and decision tree models showed that literacy was a key risk factor for cognitive dysfunction in older adults. The association between a high BMI and an increased risk of cognitive dysfunction in the present study may be because a high BMI tends to be associated with an increased risk of cardiovascular diseases such as hypertension, hyperlipidaemia, and diabetes mellitus [19–21], and these cardiovascular diseases may have an impact on the mitochondrial network system [22], which affects cerebrovascular oxygen sensing in the brain and can lead to cognitive decline. In addition, in a state of obesity, adipose tissue secretes a number of inflammatory factors, such as interleukin-6 (IL-6) and tumour necrosis factor-alpha (TNF-α) [23], which may cause an inflammatory response in the circulatory system and adversely affect brain function, thereby increasing the risk of cognitive decline. It has also been shown that obese individuals show reduced grey matter volume and thickness and increased white matter high-intensity loading, leading to grey and white matter damage, and these changes may lead to compromised functioning in various areas of the brain, including cognitive control, learning, and memory [24]. Last, we know that a high BMI is often associated with unhealthy lifestyles such as being sedentary and having chronic bad habits (e.g., tobacco and alcohol abuse) [25, 26]. These poor lifestyles may have a negative impact on brain health and increase the risk of cognitive decline.

The results of logistic regression in this study showed that older adults who had always consumed alcohol or had quit drinking alcohol had a greater risk of cognitive dysfunction than those who had never consumed alcohol. Chronic alcohol consumption can cause alcohol-related neurotoxicity, leading to damage to nerve cells, which can affect cognitive function; however, by abstaining from alcohol, the nervous system has the opportunity to recover and repair, which in turn reduces the risk of cognitive impairment [27]. Although alcohol cessation may have a protective effect on cognitive function, interindividual differences and other factors, such as age, genetic factors, and the presence of chronic diseases, which can also have an impact on cognitive function, still need to be taken into account [28]. Therefore, in addition to alcohol cessation, comprehensive health management, including a balanced diet, moderate exercise, and cognitive training, is important for reducing the risk of cognitive impairment.

### Impact of physical functioning status on cognitive dysfunction in older adults

The maintenance of balance is dependent on the balance-receptive organs of the inner ear. If these receptor organs are damaged or dysfunctional, balance regulation can be affected, which can negatively affect cognitive function. The results of this study showed that older adults with normal balance function were 0.810 times more likely to have cognitive decline than were those with impaired somatic function. In the study by Liu et al. [29], a survey of 9,006 community-dwelling older adults also showed that somatic function was an influencing factor in cognitive function. A survey of 1,386 community-dwelling older adults [30] showed that older adults with poor somatic functioning had poorer nutritional status and increased depression symptoms, leading to poorer cognitive functioning. Therefore, it is recommended that caregivers in elderly care facilities focus on older people with impaired somatic functioning, ensure their psychological well-being by increasing social support and implementing psychological interventions, and improve their cognitive functioning by personalizing meals and ensuring a good nutritional status.

Consistent with previous studies, the present study showed that ADLs are an important imaging factor influencing the onset of cognitive dysfunction in older adults. Normal ADLs can help delay or prevent the onset of cognitive dysfunction in older adults, and impaired ability to perform activities of daily living can reduce an individual’s level of cognitive function [31]. If an individual is unable to perform activities of daily living independently due to physical impairment or loss of function, problems such as social isolation, fatigue and depression can occur. These factors may further affect an individual’s cognitive abilities, such as attention, thinking flexibility and memory.

### Decision tree analysis of factors influencing cognitive dysfunction in older adults

A decision tree has a structure similar to a folded graph and is capable of extracting classification rules from irregular situations. It compares the attribute values of each internal node, determines the branches below the node, and draws classification conclusions for the leaf nodes. To make our results more credible and robust, we further performed decision tree analysis based on the CHAID algorithm. In this study, the decision tree model was used as an intuitive and interpretable way to determine that low ability to perform activities of daily living (ADLs), low scores on the Balance Test Assessment, low literacy, old age, Uyghur ethnicity, annual physical examination, and widowed status are important risk factors for cognitive impairment in older adults. In particular, activities of daily living are the most critical risk factors for the development of cognitive impairment in old age. In elderly patients with cognitive dysfunction and daily self-care deficits, the risk of cognitive dysfunction is relatively high in poor balance, especially in widowed elderly individuals, who are most likely to develop cognitive dysfunction. Therefore, based on the results of this study, it is recommended that managers take a starting point in preventing cognitive dysfunction in older adults by improving their ability to perform activities of daily living and understanding the cultural background, education and cognitive stimulation experiences of older adults. The early warning mechanism for the risk of falls among older people in the community should be gradually improved, and prevention and intervention in terms of awareness of healthy eating among older people should be strengthened to reduce the incidence of cognitive dysfunction among older people.

The results of this study indicate that Han people are more prone to cognitive impairment than ethnic minorities are. In fact, cognitive impairment is not a specific issue for a particular ethnic group or ethnicity but rather a common physical and mental health problem. The reason for this may be that the Han population is large, so the number of patients among the Han population is relatively high. Moreover, with the increasing ageing population, the risk of cognitive impairment among elderly people is increasing, and the elderly population in China is becoming more concentrated in the Han population, which may also lead to cognitive impairment being more common among the Han population. However, this does not mean that ethnic minorities are not at risk of cognitive impairment. According to the literature, Uyghur people and other ethnic minorities prefer heavy oil, salt, and sweets in their diet [32]. A high-salt and high-oil diet is closely related to the occurrence of chronic diseases, which increase the risk of chronic diseases such as hypertension, diabetes and high cholesterol among ethnic minorities, while elderly people with chronic diseases are more likely to suffer from cognitive impairment [33]. Therefore, the occurrence of cognitive impairment is a complex problem that is closely related to multiple factors, such as human genetics, environment, lifestyle, and health status, and is not directly related to ethnic identity.

The results of this study suggest that the experience of widowhood can negatively affect cognitive function in older adults, consistent with the findings of Chen et al. [34]. Widowhood can cause psychological stress and emotional distress, which can negatively affect cognitive function, and long-term emotional states such as sadness, anxiety, and depression may interfere with the normal functioning of the brain and lead to problems such as poor concentration and memory loss [35]. Second, widowhood may also cause lifestyle changes, such as changes in eating habits, sleep quality, and social activities [36]. Poor lifestyle habits can lead to physical health problems such as cardiovascular disease and metabolic disorders, which are also associated with decreased cognitive function. In addition, widowed individuals may face a lack of social support networks and a lack of intimacy and emotional support. Social activities and interpersonal relationships play important roles in the maintenance and promotion of cognitive function. Notably, widowhood itself does not necessarily lead to cognitive dysfunction. Because everyone’s situation and coping style are different, the research team recommends that older adults who have experienced widowhood reduce their risk of cognitive decline by seeking social support, maintaining a positive mindset, and maintaining a healthy lifestyle.

## Conclusions

This study is the first to investigate the risk factors for cognitive dysfunction in older adults in Northwest China. Despite some limitations, there are some valuable references. First, traditional risk factors such as physical inactivity, living alone, and Han ethnicity had a significant impact on the increased risk of cognitive dysfunction associated with older adults in Northwest China. Second, potentially modifiable risk factors such as obesity and excessive alcohol consumption had a significant effect on the increased risk of cognitive impairment among older adults in Northwest China compared with other healthy older adults. More importantly, we used a decision tree model to analyse and emphasize the role of six factors, namely, activities of daily living (ADL), total scores on the Balanced Test Assessment, educational attachment, marital status, age, ethnicity, and annual medical examination, in the management and assessment of cognitive impairment in elderly individuals in Northwest China in the future. Our study used a decision tree model, which is different from traditional statistical methods. Decision tree modelling is a simple, intuitive and practical hierarchical approach that can help health care professionals make risk-based decisions more effectively. Therefore, it is worthwhile to promote this model in future medical research. In addition, we plan to expand the sample size and introduce new indicators to assess cognitive dysfunction in elderly individuals. Through these efforts, we will further enrich and strengthen our theories for better prevention and management of this disease. This will help promote research and practice in the field of cognitive health in the elderly population and provide a more reliable basis for relevant decision-making.

### Limitations

The present study was only a cross-sectional study, which did not allow for causal interpretation, and future in-depth longitudinal studies could be conducted to further understand the trajectory of factors influencing cognitive dysfunction in older adults. Despite these limitations, our study has at least two noteworthy strengths. First, we used a large sample size, which, along with the small amount of missing data encountered, gives us enough confidence to believe that we did not miss any important information. Second, our study used a decision tree model, which possesses simple, intuitive, and hierarchical features that help health care professionals make more effective risk-based decisions.

## Data Availability

The datasets generated and analysed during the current study are not publicly available because this work is part of a larger study. The datasets are available from the corresponding author upon reasonable request.

## Abbreviations

ADLs: Activities of Daily Living
BMI: Body Mass Index
CHAID: Chi-square Automatic Interaction Detection

## References

[1] Jongsiriyanyong S, Limpawattana P (2018). Mild cognitive impairment in clinical practice: a review article. Am J Alzheimers Dis Other Dement.

[2] Nichols E, Steinmetz JD, Vollset SE, Fukutaki K, Chalek J, Abd-Allah F, Abdoli A, Abualhasan A, Abu-Gharbieh E, Akram TT, et al. Estimation of the global prevalence of dementia in 2019 and forecasted prevalence in 2050: an analysis for the Global Burden of Disease Study 2019. Lancet Public Health. 2022;7(2):e105–e125. 10.1016/S2468-2667(21)00249-8 PMC8810394 34998485

[3] Jia L, Du Y, Chu L, Zhang Z, Li F, Lyu D, Li Y, Li Y, Zhu M, Jiao H (2020). Prevalence, risk factors, and management of dementia and mild cognitive impairment in adults aged 60 years or older in China: a cross-sectional study. Lancet Public Health.

[4] Wu YT, Ali GC, Guerchet M, Prina AM, Chan KY, Prince M, Brayne C (2018). Prevalence of dementia in mainland China, Hong Kong and Taiwan: an updated systematic review and meta-analysis. Int J Epidemiol.

[5] Zhang Q, Wu Y, Han T, Liu E (2019). Changes in cognitive function and risk factors for cognitive impairment of the elderly in China: 2005–2014. Int J Environ Res Public Health.

[6] Jaroudi W, Garami J, Garrido S, Hornberger M, Keri S, Moustafa AA (2017). Factors underlying cognitive decline in old age and Alzheimer’s disease: the role of the hippocampus. Rev Neurosci.

[7] Cao L, Zhao Z, Ji C, Xia Y (2021). Association between solid fuel use and cognitive impairment: a cross-sectional and follow-up study in a middle-aged and older Chinese population. Environ Int.

[8] Litle VR (2018). Imaging and scopes? It’s bloody time for another branch in the decision tree. J Thorac Cardiovasc Surg.

[9] Rosenblatt WH, Yanez ND (2022). A decision tree approach to airway management pathways in the 2022 difficult airway algorithm of the American Society of Anesthesiologists. Anesth Analg.

[10] Yang L, Jin X, Yan J, Jin Y, Yu W, Wu H, Xu S (2016). Prevalence of dementia, cognitive status and associated risk factors among elderly of Zhejiang Province, China in 2014. Age Ageing.

[11] Wang YY, Zhang M, Wang XX, Liu S, Ding H (2022). Correlates of cognitive impairment in the elderly in China: a cross-sectional study. Front Public Health.

[12] Folstein MF, Folstein SE, McHugh PR (1975). “Mini-mental state”. A practical method for grading the cognitive state of patients for the clinician. J Psychiatr Res.

[13] Wang ZY, Zhang MY (1989). Application of simplified intelligent state check (MMSE) in Chinese version. Shanghai Psychiatry.

[14] Mahoney FI, Barthel DW (1965). Functional evaluation: the Barthel index. Md State Med J.

[15] Lawton MP, Brody EM (1969). Assessment of older people: self-maintaining and instrumental activities of Daily Living. Gerontologist.

[16] “Short Physical Performance Bat-tery (SPPB).” National Institute on Aging. https://sppbguide.com/. Accessed 9 Sept 2023.

[17] Richardson PD (1991). The “timed up & go”: a test of basic functional mobility for frail elderly persons. J Am Geriatr Soc.

[18] Livingston G, Huntley J, Sommerlad A, Ames D, Ballard C, Banerjee S, Brayne C, Burns A, Cohen-Mansfield J, Cooper C (2020). Dementia prevention, intervention, and care: 2020 report of the Lancet Commission. Lancet (London England).

[19] Nikbakht HR, Najafi F, Shakiba E, Darbandi M, Navabi J, Pasdar Y (2023). Triglyceride glucose-body mass index and hypertension risk in Iranian adults: a population-based study. BMC Endocr Disord.

[20] Ruixing Y, Jinzhen W, Weixiong L, Yuming C, Dezhai Y, Shangling P (2009). The environmental and genetic evidence for the association of hyperlipidemia and hypertension. J Hypertens.

[21] Gao S, Zhang H, Long C, Xing Z (2021). Association between obesity and microvascular diseases in patients with type 2 diabetes mellitus. Front Endocrinol.

[22] Dromparis P, Michelakis ED (2013). Mitochondria in vascular health and disease. Annu Rev Physiol.

[23] Torres-Acosta N, O’Keefe JH, O’Keefe EL, Isaacson R, Small G (2020). Therapeutic potential of TNF-α inhibition for Alzheimer’s disease prevention. J Alzheimer’s Disease.

[24] García-García I, Michaud A, Jurado M, Dagher A, Morys F (2022). Mechanisms linking obesity and its metabolic comorbidities with cerebral grey and white matter changes. Rev Endocr Metabolic Disorders.

[25] González-Gross M, Meléndez A (2013). Sedentarism, active lifestyle and sport: impact on health and obesity prevention. Nutr Hosp.

[26] Webster AJ (2022). Causal attribution fractions, and the attribution of smoking and BMI to the landscape of disease incidence in UK Biobank. Sci Rep.

[27] Wei J, Dai Y, Wen W, Li J, Ye LL, Xu S, Duan DD (2021). Blood-brain barrier integrity is the primary target of alcohol abuse. Chemico-Biol Interact.

[28] Zhang R, Shen L, Miles T, Shen Y, Cordero J, Qi Y, Liang L, Li C (2020). Association of low to moderate alcohol drinking with cognitive functions from middle to older age among US adults. JAMA Netw open.

[29] Liu Y, Gu N, Jiang L, Cao X, Li C (2021). Predicting cognitive function based on physical performance: findings from the China health and retirement longitudinal study. Aging Clin Exp Res.

[30] Wu X, Hou G, Han P, Yu X, Chen X, Song P, Zhang Y, Zhao Y, Xie F, Niu S (2021). Association between physical performance and cognitive function in Chinese community-dwelling older adults: serial mediation of malnutrition and depression. Clin Interv Aging.

[31] Chang CF, Yang RJ, Chang SF, Chou YH, Huang EW (2017). The effects of quality of life and ability to perform activities of daily living on mild cognitive impairment in older people living in publicly managed congregate housing. J Nurs Res.

[32] Li TG, Wang M (2017). [Paying attention to different health needs of different ethnic groups in process health for all program]. Zhonghua Liu Xing Bing Xue Za Zhi.

[33] Zhou B, Perel P, Mensah GA, Ezzati M (2021). Global epidemiology, health burden and effective interventions for elevated blood pressure and hypertension. Nat Rev Cardiol.

[34] Chen C, Mok VCT (2018). Marriage and risk of dementia: systematic review and meta-analysis of observational studies. J Neurol Neurosurg Psychiatry.

[35] Pink A, Krell-Roesch J, Syrjanen JA, Vassilaki M, Lowe VJ, Vemuri P, Stokin GB, Christianson TJ, Kremers WK, Jack CR (2022). A longitudinal investigation of Aβ, anxiety, depression, and mild cognitive impairment. Alzheimers Dement.

[36] Yang C, Sun X, Duan W (2021). Widowhood and life satisfaction among Chinese elderly adults: the influences of lifestyles and number of children. Front Public Health.

